# A New Measure of Mnemonic Discrimination Applicable to Recognition Memory Tests With Continuous Variation in Novel Stimulus Interference

**DOI:** 10.1002/brb3.70303

**Published:** 2025-06-26

**Authors:** Simon Léger, Christian Guinard, Selena Singh, Suzanna Becker, Jasmyn E. A. Cunningham, Martin Alda, Aaron J. Newman, Thomas Trappenberg, Abraham Nunes

**Affiliations:** ^1^ Department of Psychiatry Dalhousie University Halifax Nova Scotia Canada; ^2^ Faculty of Computer Science Dalhousie University Halifax Nova Scotia Canada; ^3^ Department of Psychology Neuroscience and Behaviour, McMaster University Hamilton Ontario Canada; ^4^ Department of Psychology and Neuroscience Dalhousie University Halifax Nova Scotia Canada

**Keywords:** mnemonic discrimination, mnemonic similarity task, MST, recognition memory

## Abstract

**Background:**

Mnemonic discrimination (MD) involves distinguishing new stimuli from highly similar memories; it is impaired in the elderly and individuals with neuropsychiatric disorders and may also probe hippocampal dentate gyrus function. Measuring MD is, therefore, highly relevant; however, the gold‐standard MD test, the mnemonic similarity task (MST), is rarely used in clinical research. Thus, it would be useful to develop a novel MD index applicable to recognition memory tasks that are commonly used in clinical research. The present study develops such a measure and demonstrates its convergent validity with the gold‐standard MD index from the MST.

**Methods:**

We derived participant‐level indices of MD (*λ*) and overall recognition memory performance (Δ) by fitting a logistic function to the relationship between stimulus interference and the probability of classifying a stimulus as novel. We then applied these novel measures to two independent MST datasets (*N* = 18; *N* = 67) and to simulated MST data. We used linear mixed‐effects model to test whether (1) *λ* predicts the MST's MD measure, the *lure discrimination index* (LDI), and (2) Δ predicts the MST's overall recognition memory index (REC).

**Results:**

*λ* predicted LDI (*β* = 0.76, 95% CI [0.62, 0.91], *p *< 0.001) but not REC (*β* = 0.06, 95% CI [−0.03, 0.15], *p* = 0.197), while Δ predicted REC (*β* = 0.93, 95% CI [0.83, 1.02], *p *< 0.001) but not LDI (*β* = −0.06, 95% CI [−0.20, 0.09], *p* = 0.438). The *λ* and Δ indices were statistically independent, although simulations with synthetic data suggest that MD measurement may be compromised if overall recognition memory performance is impaired.

**Conclusion:**

We have developed a novel measure of MD applicable to two‐choice recognition memory tasks that use stimuli with continuously varying degrees of similarity. Future studies should further validate this measure using large clinical datasets that include both MD and other recognition memory tasks.

## Graphical abstract

Novel, widely applicable mnemonic discrimination indices show convergent and divergent validity with the standard mnemonic similarity task indices.

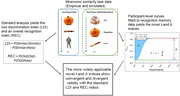

## 1 Introduction

Mnemonic discrimination (MD) is the ability to distinguish new stimuli from highly similar memories and may probe dentate gyrus functioning (Bakker et al. 2008; Baker et al. 2016; Klippenstein et al. 2020; Berron et al. 2016; Wang et al. 2023; Marlatte et al. 2024). Poor MD is associated with aging (Yassa et al. 2011; Stark, et al. 2013; Ilyés, Paulik, and Keresztes 2024) and several neuropsychiatric conditions (Baker et al. 2016; Stark et al. 2013; Das et al. 2014; Brock Kirwan et al. 2012, Hanert, Pedersen, and Bartsch 2019; Bakker et al. 2012; Déry et al. 2013; Shelton and Kirwan 2013; Han et al. 2020), may be improved by lithium in lithium‐responsive bipolar disorder patients (Madanlal et al. 2024) and may be able to identify people at risk of schizophrenia (İmamoğlu et al. 2023). MD assessment is, therefore, clinically relevant; however, our ability to study MD in clinical populations is currently limited by a few available samples of patient data using the gold‐standard measure of MD, the *mnemonic similarity task* (MST) (Stark, Kirwan, and Stark 2019). Conversely, recognition memory testing in clinical research often utilizes standard clinical batteries such as verbal learning tests (e.g., the California Verbal Learning Test [CVLT]) (Delis 2000), for which large samples exist (Kennedy et al. 2024). Enabling measurement of MD from these tests could facilitate highly powered studies of MD in neuropsychiatric populations to examine the extent of MD's clinical importance at a low cost. However, to our knowledge, there is no validated approach to examining MD using these standard recognition memory paradigms.

The present study thus aims to develop a novel approach to quantify MD performance using recognition memory paradigms other than the MST. The MST is a delayed recognition memory paradigm in which participants must study a set of images in a *study list* to then identify repeated images, here called “old” images, in a subsequent *test list* (see Figure S1). Importantly, the test list contains highly distinct images, here called “foils,” and images that are similar, but not identical, to the old images, here called “lures” (Stark, Kirwan, and Stark 2019). Critically, the MST has a *categorical* separation between old, lure, and foil stimuli, which facilitates the measurement of overall recognition memory performance (called *REC*) and MD via the *lure discrimination index* (LDI). The REC measure is quantified as the probability that an old image is classified as old minus the probability that a foil image is misclassified as old. The LDI is quantified as the probability that a lure image is classified as “similar” minus the probability that a foil image is misclassified as “similar.” Other analytical methods have been proposed for the MST, but they all require clear categorical distinctions between which images in the test set are lures and foils (Lee and Stark 2023; Loiotile and Courtney 2015). However, memory tests commonly used in clinical research typically feature test stimuli whose degree of similarity varies in a more continuous fashion rather than having clear categorical distinctions between foils and lures. This prevents the LDI and REC measures from being directly applied to these common recognition memory tests.

We, therefore, developed a novel approach to quantify MD in two‐choice recognition memory tasks that have continuous variation in stimulus dissimilarities, derived from a general mathematical formulation of MD paradigms. We show that our approach closely tracks the gold‐standard measures, REC and LDI, as computed from two MST studies, demonstrating both convergent and divergent validity. Finally, we generated simulated MST data to examine conditions that may moderate the performance of our novel approach.

## 2 Methods—Empirical Validation

### 2.1 Mathematical Generalization of Mnemonic Discrimination Paradigms

To develop a novel set of indices for MD, we first sought to develop a task‐agnostic generalization of MD paradigms to extract the task components necessary for MD measurement. Let X represent a set of stimuli used in a recognition memory task, which is a random variable defined on a discrete space {1,2,…,K}. Let $x_{i}$ be the i’th realization of X, sampled with replacement.

A recognition memory experiment consists of an initial study phase in which an agent is supplied with a sequence of N items of X, denoted $\bar{X}=\;\left\{x_{1},x_{2},\text{…}\;,x_{N}\right\}$. For some index n, where 1<n<N, we can divide the list of items $\bar{X}$ into a study and test list, calling ${\bar{X}}_{S}=\;\left\{x_{1},\text{…}\;,x_{n}\right\}$ the *study list*, and ${\bar{X}}_{T}=\left\{x_{n+1},x_{n+2},\text{…}\;,x_{N}\right\}$ the *test list*. Given this division, let $Y^{*}=\left\{y_{i}^{*}\;:\;i\;\in \;1,2,\text{…}\;,\;N\;-\;n\right\}$ be a vector denoting whether item *x_n + _*_i_ is novel; $y_{i}^{*}=I\;I\left[x_{n+i}\;\notin \;{\bar{X}}_{S}\right],$ which takes a value of 1 if the argument is true, and a value of 0 otherwise. When $y_{i}^{*}=1$, then *x_n + _*_i_ is “new” (either a “lure” or “foil”), and when $y_{i}^{*}=0$, it is known as a “target.”

Let $y_{i}$ be the agent's estimate of $y_{i}^{*}$ (i.e., its prediction of whether $x_{n+i}\;\notin \;{\bar{X}}_{S}$). We assume that $y_{i}$ is a continuous or discrete prediction generated by a function $f_{\theta }\left(x_{n+i};\;{\bar{X}}_{S}\right)$ governed by some parameters θ that characterize the agent, as well as the list of items studied.

A recognition memory paradigm becomes an MD paradigm when we consider how the probability of identifying test list stimulus *x_n + _*_i_ as novel, which here we will denote as $p_{\theta }$(*x_n + _*_i_), varies in relation to the degree of similarity between *x_n + _*_i_ and the most similar stimulus in the study list ${\bar{X}}_{S}$. If we let $d(x_{n+i},{\bar{X}}_{S})$ represent some measure of perceptual or semantic “dissimilarity” between the item *x_n + _*_i_ and study list ${\bar{X}}_{S}$, such that $d(x_{n+i},{\bar{X}}_{S})=0$ implies that *x_n + _*_i_ is an old item in the study list (no dissimilarity), then $p_{\theta }\left(x_{n+i}\right)$ should increase monotonically with respect to $d(x_{n+i},{\bar{X}}_{S})$. That is, as the stimulus *x_n + _*_i_ becomes more distinct from the stimuli in the study list, the more likely the participant is to classify it as novel. For an individual with excellent MD ability, where their recognition memory system is highly sensitive to even small differences between old and new stimuli, *p*(*x_n + _*_i_) approaches its maximal value for even small values of $d(x_{n+i},{\bar{X}}_{S})$.

### 2.2 Novel Measures of Recognition and Mnemonic Discrimination Performance

**FIGURE 1:**
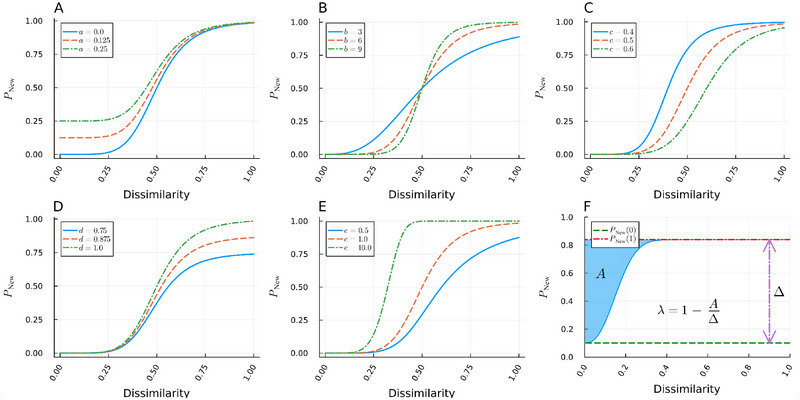
Consequences of altering parameters on the five‐parameter logistics function. (A) Parameter *a* primarily corresponds to the lower asymptote, which will mostly influence the probability of misclassifying an old image as new. (B) Parameter *b* changes the steepness of the curve. (C) Parameter *c* shifts the curve horizontally. (D) Parameter *d* primarily corresponds to the upper asymptote, which will mostly influence the probability of classifying the most distinct new image as new. (E) Parameter *e* adjusts the asymmetry of the curve. (F) Example of a fitted curve. The dashed green line shows *P*_NEW_(0), representing the probability of classifying an old image as new. The dot‐dashed red line shows *P*_NEW_(1), representing the probability of classifying *the most distinct* (semantically dissimilar) new image as new. The difference between *P*_NEW_(1) and *P*_NEW_(0) defines an overall measure of recognition memory performance, Δ. The *λ* index of mnemonic discrimination is calculated by examining the degree to which *P*_NEW_ declines with increasingly similar stimuli, specifically by calculating the inverse of the area between the curve and its upper asymptote and dividing it by Δ.

For each participant, let *P*_NEW_(*dissimilarity*) be a performance curve representing the probability of classifying stimuli as new, which we assume strictly increases with respect to dissimilarity. Indexing recognition in such a curve involves calculating the difference between *P*_NEW_ (Bakker et al. 2008), correct identification of the most dissimilar items, and *P*_NEW_(0), incorrect identification of “old” items as being novel. We call this the recognition index Δ = *P*_NEW_(1) − *P*_NEW_(0). To index MD, we need to capture how far *P*_NEW_(*dissimilarity*) deviates from *P*_NEW_(1), in relation to dissimilarity. For instance, perfect MD would imply that *P*_NEW_(*dissimilarity*) ≈ *P*_NEW_ (Bakker et al. 2008) for all dissimilarity values greater than 0. The MD index should be maximal in those cases. Conversely, the MD index should be low if it only reaches its maximum value at high values of dissimilarity. We modelled the relationship *P*_NEW_(dissimilarity) using the following five‐parameter sigmoidal function,

$$P_{\mathrm{NEW}}\left(\mathrm{dissimilarity}\right)=d+\left(a-d\right)/{\left(1+{\left(\mathrm{dissimilarity}/c\right)}^{b}\right)}^{e},$$

which is graphically depicted in Figure 1, and which we fit to individual participants' trial‐by‐trial data. Parameter *a* primarily corresponds to the lower asymptote, which will mostly influence the probability of misclassifying an old image as new, parameter *b* changes the steepness of the curve, parameter *c* shifts the curve horizontally, parameter *d* primarily corresponds to the upper asymptote, which will mostly influence the probability of classifying the most distinct new image as new, and finally, parameter *e* adjusts the asymmetry of the curve.

With a performance curve representing *P*_NEW_(*dissimilarity*), we can calculate its deviation from *P*_NEW_ (Bakker et al. 2008) by taking the area between the performance curve and its maximum. We then scale the output by Δ to reduce the collinearity of the two measures. Our MD index, denoted as *λ*, is thus defined as follows: *λ* = 1 *− A*/Δ, where *A* = *P*_NEW_(1) − ∫_0_^1^ *P*_NEW_(*x*) *dx* (Figure 1F)*. A* is the integral defining the area between the sigmoidal function *P*_NEW_(dissimilarity) and the maximal value of *P*_NEW_(1), the latter of which represents the correct identification rate of the most distinct novel items.

### 2.3 Experimental Validation

Using two MST datasets, we sought to verify that *λ* tracks LDI, and that Δ tracks REC. The first dataset from Lee and Stark (2023) included data collected from 21 participants (13 female, mean age: 21) using the three‐way classification (old–similar–new) MST, studying 128 images and testing on 192 images (64 old, 64 new, 64 lures). The second dataset from Wahlheim et al. (2022) included data from 72 participants (36 “younger,” mean age: 22.21; 36 “older,” mean age: 69.82) performing a similar MST with 72 study images and 108 test images (36 old, 36 new, 36 lures). Participants were recruited from the University of California at Irvine and the Greensboro, North Carolina community, respectively, with ethics approvals and data available online. In both of these studies, each lure image had an associated lure bin value from 1 to 5, indicating its similarity to a test set image (1 being highly similar, 5 being least similar). Lure bin sets have previously been empirically determined for the MST by grouping image pairs with similar false alarm rates, where Bin 1 images are most often misclassified as old, and Bin 5 images are least often misclassified (Stark et al. 2013; Lacy et al. 2011). Data from three participants from Dataset 1 and five from Dataset 2 were excluded due to excessive missing or random responses. A participant short of the exclusion threshold was excluded in an additional analysis (see Figure S3 and Tables S1–S3 for more details).

### 2.4 Statistical Analysis

For our analyses, we translated lure bins into measures of dissimilarity. We assume the lure bin of new trials to be 6 and old trials to be 0. For each study, the data were preprocessed such that the ordinal lure bins were standardized into a “dissimilarity index” that ranged between 0 (old stimuli) and 1 (most dissimilar). This was done by dividing each trial's lure bin value by the maximum of 6. This facilitated the computation of $p_{\theta }$(*x*) for some stimulus *x*, representing the probability that the participant will identify the test image as novel.

To model the influence of dissimilarity on responses, we fit the five‐parameter logistic function to each participant's combination of stimulus dissimilarity values and old/new/similar responses (participant responses were coded as 0 for “old” responses and 1 for “similar” and “new” responses). This was done using nonlinear least squares in the *LsqFit.jl* package for the Julia programming language (https://github.com/JuliaNLSolvers/LsqFit.jl). From these fitted curves, we extracted the *λ* and Δ measures for each participant. Finally, we also calculated the original MST LDI and REC scores for each participant.

To demonstrate our measure's convergent validity with the MST, we examined whether *λ* and Δ explained variation in the LDI and REC, respectively. To demonstrate divergent validity, we evaluated whether Δ was not associated with the LDI, and that *λ* was not associated with the REC. Finally, we checked for collinearity between *λ* and Δ. These analyses were conducted by fitting the following linear mixed‐effects models to participant data using the *lme4* package for the R programming language (Bates et al. 2015), presented here in R syntax:

(1) $$\mathrm{LDI}∼\lambda +\Delta +\left(1|\mathrm{Study}\right)$$

(2) $$\mathrm{REC}∼\lambda +\Delta +\left(1|\mathrm{Study}\right)$$

(3) $$\lambda ∼\Delta +\left(1|\mathrm{Study}\right)$$

### 2.5 Sensitivity Analyses

Since many recognition tests are not designed to introduce high interference as in the MST, we sought to examine the performance of our measures in situations when most test list items are highly dissimilar from old items. This was done by reanalyzing the Lee and Stark (2023) dataset after excluding lure trials with lure bins of 3 or below (39 trials) (see Supporting Information; Table S4).

Given that many memory tests lack item dissimilarity measures, we evaluated a more widely applicable dissimilarity measure using the Wahlheim et al. (2022) dataset by employing deep neural network models. Briefly, we used a model pretrained on natural images that enables the transformation of raw images into embeddings that represent images' perceptually salient high‐level features. The embeddings of the MST images can be compared to one another to obtain distances that can represent the perceptual dissimilarity of the two images (see Figures S2 and S4 and Table S5 for more details).

### 2.6 Results—Empirical Data

**FIGURE 2:**
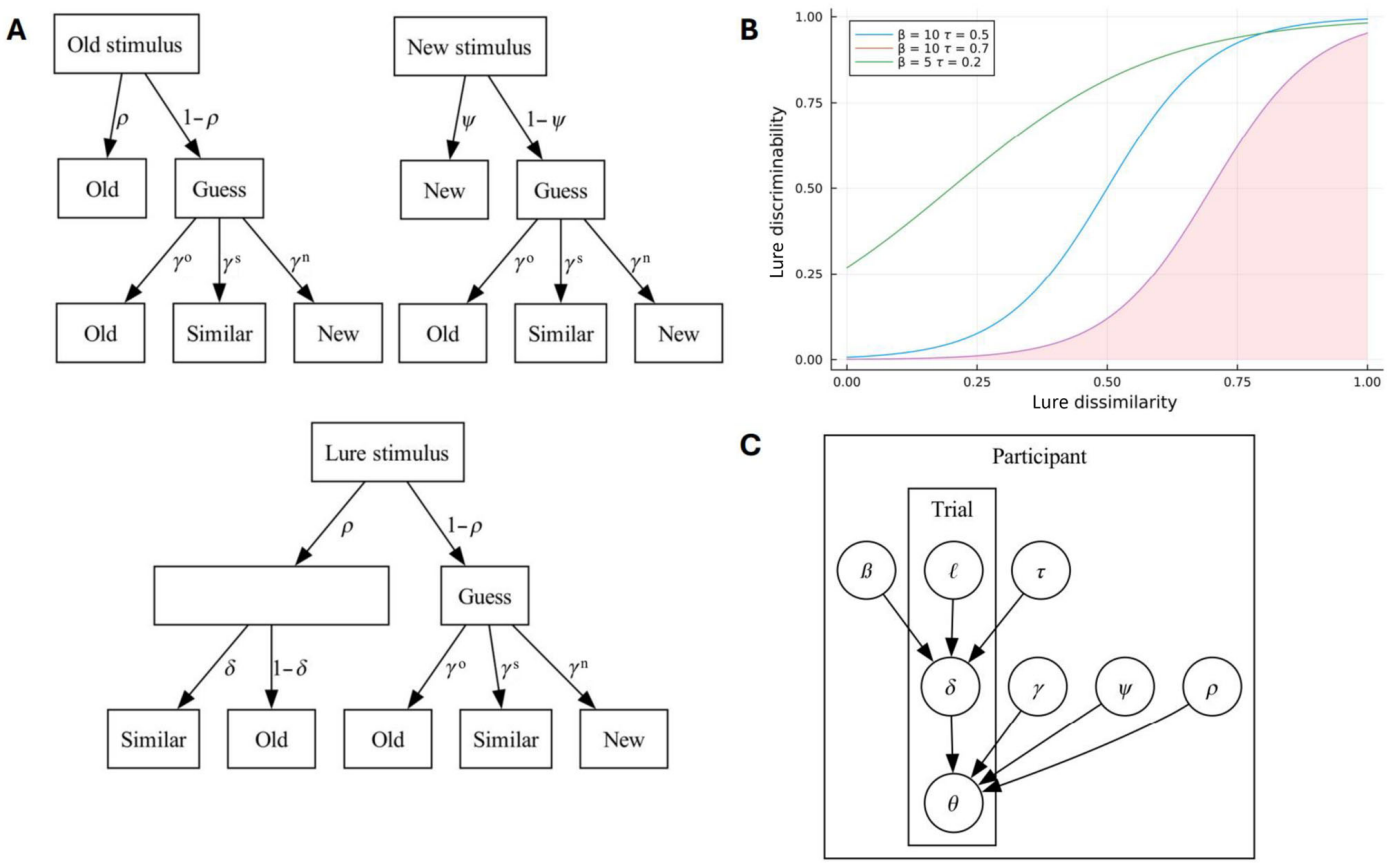
Pairwise comparisons of the original mnemonic similarity task (MST) measures and our novel measures. Colors indicate the study that data are taken from: Lee and Stark 2023 in blue, Wahlheim et al. 2022 in red. (A) The MST's lure discrimination index (LDI) and the novel *λ* measure. (B) The MST's recognition measure (REC) and the novel Δ measure. (C) The novel Δ and the novel *λ* measure. (D) The MST's LDI and the novel Δ measure. (E) The MST's REC and the novel *λ* measure.

Table 1 shows the results of our mixed‐effects modelling approach verifying concordance between the original MST performance indices and our novel *λ* and Δ measures. Our *λ* measure showed a statistically significant association with the MST LDI index (*β* = 0.76, 95% CI [0.62, 0.91], *p *< 0.001; Table 1 and Figure 2A), while not being significantly associated with the MST REC (*β* = 0.06, 95% CI [−0.03, 0.15], *p* = 0.197; Table 1 and Figure 2E). Similarly, our Δ measure showed significant association with the MST REC (*β* = 0.93, 95% CI [0.83, 1.02], *p *< 0.001; Table 1 and Figure 2B), while not being significantly associated with the MST LDI (*β* = −0.06, 95% CI [−0.20, 0.09], *p* = 0.438; Table 1 and Figure 2D). The fixed effects coefficient of our linear mixed model did not indicate a statistically significant association between *λ* and Δ (*β* = 0.21, 95% CI [−0.01, 0.43]; *p* = 0.061; Table 1 and Figure 2C). The model exhibited a marginal *R* ^2^ of 0.042 and a conditional *R* ^2^ of 0.099, with an ICC of 0.06, suggesting consistency across the two studies.

**TABLE 1:** Mixed‐effects model results of Δ predicting *λ* scores, of *λ* and Δ predicting original MST LDI scores, and of *λ* and Δ predicting the original MST REC scores. P‐values reaching statistical significance are bolded.

Predictors	Estimates	CI	*p*
Model 1: *λ* ∼ Δ + (1|study)			
(Intercept)	0.08	−0.35 to 0.50	0.713
Δ	0.21	−0.01 to 0.43	0.061
Random effects			
*σ* ^2^	0.96		
*τ*_00study_	0.06		
ICC	0.06		
*N*_study_	2		
Observations	85		
Marginal *R* ^2^/Conditional *R* ^2^	0.042/0.099		
Model 2: LDI *∼ λ* + Δ + (1|study)			
(Intercept)	−0.00	−0.14 to 0.14	1.000
*λ*	0.76	0.62 to 0.91	**< 0.001**
Δ	−0.06	−0.20 to 0.09	0.438
Random effects			
*σ* ^2^	0.44		
*τ*_00study_	0.00		
Marginal *R* ^2^/Conditional *R* ^2^	0.565/NA		
Model 3: REC *∼ λ* + Δ + (1|study)			
(Intercept)	0.05	−0.18 to 0.28	0.658
*λ*	0.06	−0.03 to 0.15	0.197
Δ	0.93	0.83–1.02	**< 0.001**
Random effects			
*σ* ^2^	0.16		
*τ*_00study_	0.02		
ICC	0.11		
Marginal *R* ^2^/Conditional *R* ^2^	0.830/0.849		

### 2.7 Methods—Synthetic Data Experiments

#### 2.7.1 Synthetic Data Generation

We applied our MD measure to simulated MST data to examine whether (1) *λ* agrees with the LDI in edge‐case scenarios under which *λ'*s accuracy may break down, and (2) both *λ* and LDI agree with an underlying ground truth MD measure. Synthetic MST data were generated using an established multinomial processing tree approach (Lee and Stark 2023).

**FIGURE 3:**
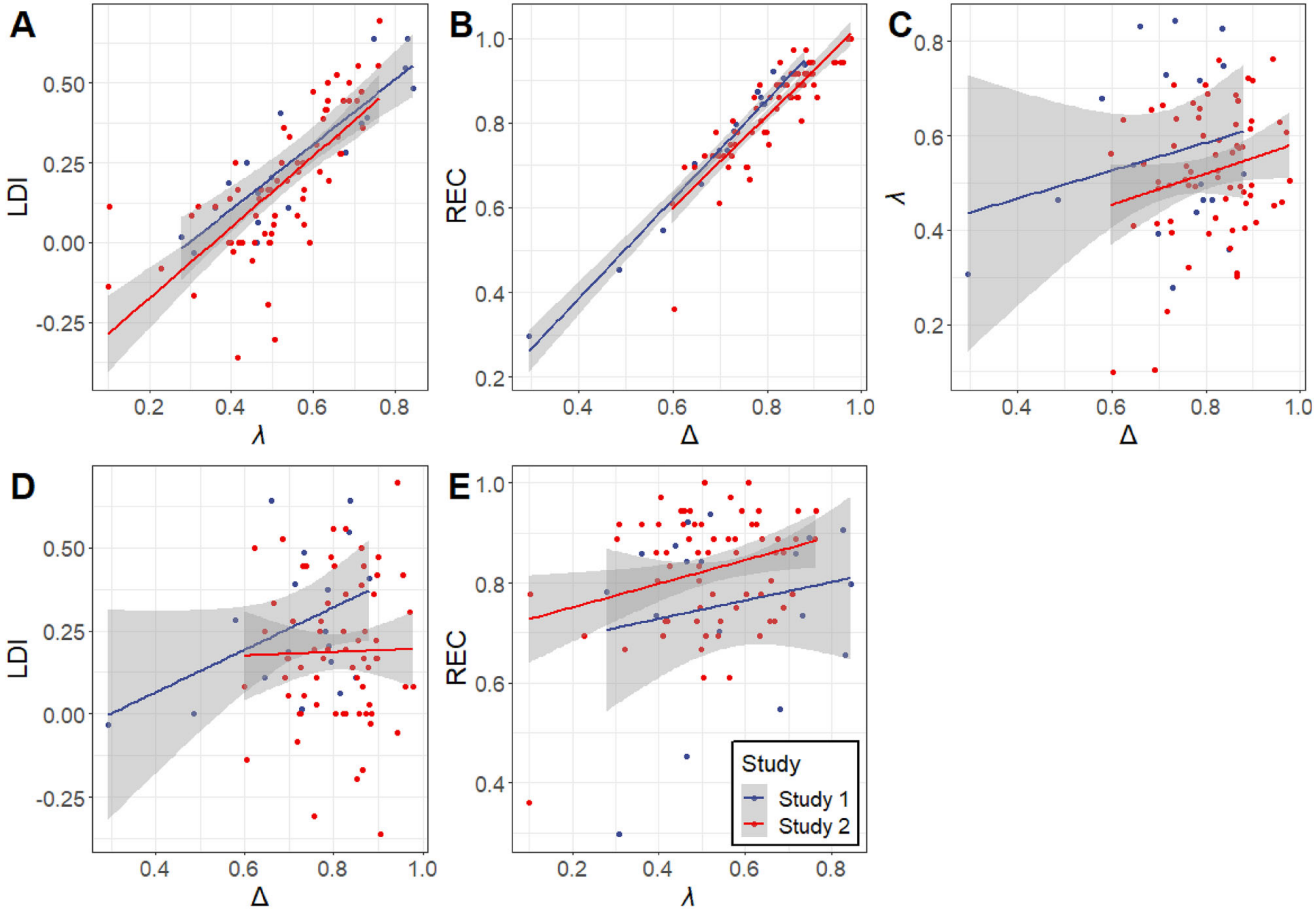
Summary plots of the synthetic data experiment methodology. (A) Visual depiction of the synthetic agent's decision trees on a test trial. Each simulated agent has set values of *ρ*, *γ*, *ψ*, *β*, and *τ* that dictates the decisions the agents take when presented with either an old, new, or lure stimulus. The *β* and *τ* parameters, alongside the relevant stimulus' lure similarity, *ℓ*, govern the *δ* parameter used in discriminating lure stimulus. (B) Graphical depiction of the two‐parameter logistic function with sample parameters used to characterize the relation between lure discriminability (*δ*) and lure dissimilarity (*ℓ*) for each simulated synthetic agent. The *β* parameter adjusts the steepness of the curve, while the *τ* parameter shifts the curve horizontally. The shaded area beneath the red curve corresponds to that curve's ground truth *λ*. (C) Plate diagram summary of the synthetic data experiment. At the participant level, each agent is characterized by the parameters *ρ*, *γ*, *ψ*, *τ*, and *β*. The parameters *τ* and *β*, together with the specific lure trial's lure similarity level *ℓ*, determine the agent's lure discriminability at that trial. Given these parameters, each agent at each trial then extracts values of $\theta^{O}$, $\theta^{S}$, and $\theta^{N}$, where $\theta^{O}+\theta^{S}+\theta^{N}=1$. These *θ* values represent the probability that the agent will determine the specific trial stimulus as being either old, new, or similar, of which the determination itself is represented graphically as the decision node.

In our model, synthetic agents respond to stimuli categorized as old, new, or lure based on predefined probabilities (graphically depicted in Figure 3A ). For non‐lure trials, agents have a probability *ρ* of recognizing an old stimulus as old and a probability *ψ* of recognizing that a new stimulus item was not previously studied. For a lure trial, agents have a probability *ρ* of recognizing the related studied item, indicating familiarity, after which they may correctly discriminate at a probability $\delta_{l}$ that the lure is similar, rather than old. If any recognition fails, the agent guesses its category—old, similar, or new—based on probabilities $\gamma^{O}$, $\gamma^{S}$, and $\gamma^{N}$, respectively, where $\gamma^{O}+\gamma^{S}+\gamma^{N}=1$.

The agents' probability δ of discriminating lures from studied items depends on the level of similarity ℓ (represented as a value between 0 and 1) of the lure stimuli to a studied item in a manner that is unique to each agent. To this end, we implemented an extension of the multinomial processing tree as per Lee and Stark (2023) such that each agent has a model of the relationship between discriminability (δ) and lure similarity (*ℓ*) for lure stimuli only (see Figure 3B for a graphical depiction of the relationship):

$$\delta_{\ell }=\frac{1}{1+\exp\left\{-\beta \left(\ell \;-\;\tau \right)\right\}}\;$$

where 0<ℓ<1.

This curve characterizes how discriminability increases as lure dissimilarity increases. The *β* parameter adjusts the steepness of the curve, while the *τ* parameter shifts the curve horizontally. In this synthetic experiment, the above‐described participant‐level relation between discriminability and lure dissimilarity contains the relevant information about MD that both LDI and the novel *λ* measure aim to extract. We characterize this information as the area under the $\delta_{\ell }\;$ curve, referred to as ground truth *λ*.

Given the above structure, graphically summarized in Figure 3C, we simulated 1500 synthetic agents that were each assigned random values between 0 and 1 of *ρ*, *γ*, *ψ*, and *τ*, and a random value between 0 and 50 for *β*. Each agent is then given a simulated test list that contains 16 old, 16 new, and 16 lure stimuli (with a uniform distribution of each possible lure similarity value). For each simulated stimulus, agents make discrete decisions on whether the stimuli are old, new, or similar.

#### 2.7.2 Statistical Analysis—Synthetic Data

Each synthetic agent's LDI and REC scores were calculated in the same manner as with the empirical data. To calculate our novel indices, we used each synthetic agent's trial responses and the trials' dissimilarities to fit the previously described five‐parameter logistic function to the synthetic participant‐level data. The fitted functions were then used to extract each agent's *λ* and Δ indices. We describe associations between simulation parameters and the novel indices in Supporting Information (see Figures S6 and S7).

We considered that poor overall recognition could impact the degree to which the *λ* index could track both LDI and the ground truth *λ*. To this end, we further examined these relations by dividing the dataset into subsets based on the agents' overall recognition. We conducted an additional analysis of these relations where the data only included synthetic agents with a Δ index of at least 0.6 (a value that unexcluded human participants generally exceed in the MST [Lee and Stark 2023]). We also progressively restricted the dataset to only those agents with increasing Δ levels while observing the fit of the linear mixed‐effects models.

#### 2.7.3 Results—Synthetic Data

**FIGURE 4:**
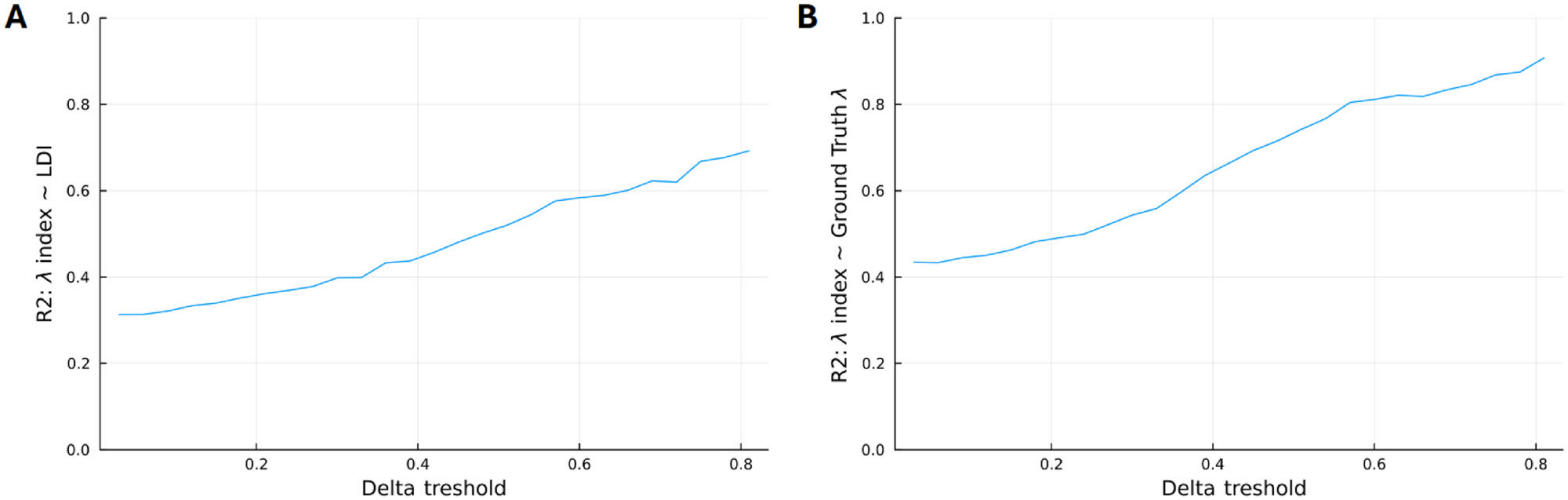
*R* ^2^ values of the relationships between the synthetic agent's *λ* index and (A) the MST's lure discrimination index (LDI), and (B) the ground truth *λ* calculated from the synthetic agent's two‐parameter logistic function. Models were subsetted to only include synthetic agents with a Δ index above the specified Δ thresholds.

Synthetic data experiments confirmed a strong association between the Δ index and REC, exhibiting an *R* ^2^ of 0.923 (see Figure S5 and Table 2). The association between the *λ* index and LDI was initially moderate but statistically significant in the full dataset (*β* = 0.55, 95% CI [0.51, 0.59], *p *< 0.001). However, this association strengthened when recognition memory performance increased (at Δ scores > 0.6: *β* = 0.76, 95% CI [0.71, 0.81], *p *< 0.001; Figure 4A). The relationship between the *λ* index and ground truth *λ* similarly improved with higher Δ thresholds. The synthetic analysis additionally confirmed the lack of a significant direct relationship between *λ* and Δ in both the full dataset and a subset with higher Δ scores (Table 2).

**TABLE 2:** Linear model results of synthetic data experiment with and without the removal of low Δ synthetic participants. P‐values reaching statistical significance are bolded.

Model: LDI *∼ λ* + Δ				Model: LDI ∼ *λ* + Δ, subset: Δ ≥ 0.6			
Predictors	Estimates	CI	*p*	Predictors	Estimates	CI	*p*
(Intercept)	0.00	−0.04 to 0.04	1.000	(Intercept)	0.00	−0.04 to 0.04	1.000
*λ*	0.55	0.51–0.59	**< 0.001**	*λ*	0.76	0.71–0.81	**< 0.001**
Δ	0.24	0.20–0.28	**< 0.001**	Δ	0.12	0.07–0.17	**< 0.001**
Observations 1488				Observations 609			
Marginal *R* ^2^/Conditional *R* ^2^: 0.352/0.352				Marginal *R* ^2^/Conditional *R* ^2^: 0.599/0.597			

Model: Ground truth *λ ∼ λ* + Δ				Model: Ground truth *λ* ∼ *λ* + Δ, subset: Δ ≥ 0.6			
Predictors	Estimates	CI	*p*	Predictors	Estimates	CI	*p*
(Intercept)	−0.00	−0.04 to 0.04	1.000	(Intercept)	0.00	−0.03 to 0.03	1.000
*λ*	0.64	0.60–0.68	**< 0.001**	*λ*	0.90	0.87–0.94	**< 0.001**
Δ	0.04	0.00–0.08	**0.043**	Δ	0.03	−0.01 to 0.06	0.154
Observations 1488				Observations 609			
Marginal *R* ^2^/Conditional *R* ^2^: 0.409/0.408				Marginal *R* ^2^/Conditional *R* ^2^: 0.812/0.811			

Model: REC *∼ λ* + Δ				Model: REC ∼ *λ* + Δ, subset: Δ ≥ 0.6			
Predictors	Estimates	CI	*p*	Predictors	Estimates	CI	*p*
(Intercept)	−0.00	−0.01 to 0.01	1.000	(Intercept)	−0.00	−0.04 to 0.04	1.000
*λ*	0.00	−0.01 to 0.02	0.805	*λ*	−0.12	−0.16 to 0.08	**< 0.001**
Δ	0.96	0.95–0.97	**< 0.001**	Δ	0.85	0.81–0.89	**< 0.001**
Observations 1488				Observations 609			
Marginal *R* ^2^/Conditional *R* ^2^: 0.923/0.923				Marginal *R* ^2^/Conditional *R* ^2^: 0.740/0.739			

Model: *λ ∼* Δ				Model: *λ* ∼ Δ, subset: Δ ≥ 0.6			
Predictors	Estimates	CI	*p*	Predictors	Estimates	CI	*p*
(Intercept)	−0.00	−0.05 to 0.05	1.000	(Intercept)	0.00	−0.08 to 0.08	1.000
Δ	−0.03	−0.08 to 0.02	0.207	Δ	0.01	−0.07 to 0.09	0.845
Observations 1488				Observations 609			
Marginal *R* ^2^/Conditional *R* ^2^: 0.001/0.000				Marginal *R* ^2^/Conditional *R* ^2^: 0.000/−0.002			

## 3 Discussion

Our novel measure of MD, *λ*, enables the study of MD in recognition memory tasks without categorical distinctions between “lures” and “foils” in test lists. We demonstrated both convergent and divergent validity for our new measures (Carlson and Herdman 2012), where *λ* was significantly associated with MST LDI but not with MST REC, and Δ was significantly associated with MST REC but not with MST LDI. This, in combination with the fact that *λ* and Δ are uncorrelated, suggests that these measures provide valid and well‐separated markers of MD (*λ*) and overall recognition memory performance (Δ). Synthetic experiments further confirmed these associations and also highlighted that the relationship between *λ* and MST LDI is stronger if an agent's Δ index is high, suggesting that MD may be difficult to measure in participants with poor overall recognition.

Future investigations should consider applying our MD measure to large existing clinical datasets that contain responses from verbal recognition memory tests such as the CVLT (Kennedy et al. 2023) in addition to MST data to assess concordance between our MD and REC measures on the MST versus other widely used recognition memory tasks. Verbal learning tests are important since impairments in this domain are common in clinical populations (Cullen et al. 2016) and are predictive of functional outcomes (Depp et al. 2012; Tse et al. 2014). Harnessing data from established tools could facilitate understanding MD in clinically diverse and large sample populations, improving generalizability. It may also be useful for future studies to evaluate ways to adapt our method of analysis to forced‐choice recognition tests.

Although common recognition memory tests are not explicitly designed to elicit high false alarm rates, variations in dissimilarity within these tests can still reveal information about MD, conditional upon an absence of ceiling effects. We demonstrated that our MD measurement approach was robust to removing the most similar lures from the test set in MST data. Specifically, we found that despite excluding the 60% most similar lures (i.e., those with the highest false alarm rates) in the analysis, the *λ* index continued to show convergent validity with the MST's LDI among the participants in the Lee and Stark (2023) dataset.

Applications to other tests without a built‐in measure of dissimilarity will require specific adaptations depending on the stimulus type employed. In the present study, we used the MST's provided ordinal dissimilarity measures to demonstrate convergent and divergent validity. However, convergent and divergent validity was still demonstrated using an alternative continuous dissimilarity measure calculated by extracting the high‐level abstract representations of the images from a deep‐learning model pretrained on natural images. Indeed, continuous dissimilarity between image representations in this abstract space may be more reflective of perceptually relevant differences between images. This method of deriving dissimilarity to index MD from a deep neural network model was also successfully applied to continuous visual memory test data in another study (Madanlal et al. 2024). Similar approaches may also be applied to verbal recognition memory tasks using language models to reflect semantic dissimilarity.

A limitation of the current study is the inclusion of only two real‐world datasets, both of which had relatively small sample sizes, due to a limited number of freely available MST datasets. Given this, our novel analytical approach should enable analysis of other existing recognition memory datasets, which may now be used to understand MD performance as well. Despite this limitation, our estimates were consistent across the datasets utilized. Nevertheless, as a further confirmatory step, future studies should evaluate our approach on additional MST data to further test the convergent and divergent validity of *λ* and Δ.

## 4 Conclusion

Our introduction of the *λ* index of MD offers an alternative for analyzing MD abilities using data from traditional recognition memory tasks that eliminates the need for categorical distinctions in participants' responses between lures and foils. Our findings demonstrate the *λ* index's convergent and divergent validity, where our *λ* measure was significantly associated with the established MST LDI but not with MST REC. The *λ* index has the potential to be a more widely applicable measure of MD. For example, our measure may be able to examine MD in large datasets using recognition memory paradigms from clinical samples, allowing for a more comprehensive understanding of MD in these populations.

## Author Contributions

**Simon Léger**: conceptualization, formal analysis, software, methodology, writing–review and editing, writing–original draft, visualization, validation, data curation, investigation. **Christian Guinard**: writing–review and editing, software, visualization, methodology, conceptualization. **Selena Singh**: conceptualization, writing–review and editing. **Suzanna Becker**: writing–review and editing. **Jasmyn E. A. Cunningham**: writing–review and editing. **Martin Alda**: writing–review and editing. **Aaron J. Newman**: writing–review and editing. **Thomas Trappenberg**: writing–review and editing. **Abraham Nunes**: writing–review and editing, project administration, methodology, funding acquisition, writing–original draft, conceptualization, supervision, resources, data curation, investigation, validation, software.

## Peer Review

The peer review history for this article is available at https://publons.com/publon/10.1002/brb3.70303.

## Supporting information



Supporting Information

Supporting Information

## Data Availability

The data that support the findings of this study are available in the Supporting Information of this article.
