# Supplementary material for: A New Measure of Mnemonic Discrimination Applicable to Recognition Memory Tests With Continuous Variation in Novel Stimulus Interference
Source: Brain Behav. 2025 Jun 26;15(6):e70303. doi: 10.1002/brb3.70303 (PMC12198479; doi:10.1002/brb3.70303)
Supplement: Supplementary file 1 — Supporting Information [file BRB3-15-e70303-s001.docx]

# Supplementary Materials for *A New Measure of Mnemonic Discrimination Applicable to Recognition Memory Tests with Continuous Variation in Novel Stimulus Interference*

Simon Léger [1,†], Christian Guinard [1,2,†], Selena Singh [3], Suzanna Becker [3], Jasmyn E.A.Cunningham [1], Martin Alda [1], Aaron J. Newman [4], Thomas Trappenberg [2], and Abraham Nunes [1,2,*]

[1] Department of Psychiatry, Dalhousie University, Halifax, Nova Scotia, Canada

[2] Faculty of Computer Science, Dalhousie University, Halifax, Nova Scotia, Canada

[3] Department of Psychology, Neuroscience & Behaviour, McMaster University, Hamilton, Ontario, Canada

[4] Department of Psychology and Neuroscience, Dalhousie University, Halifax, Nova Scotia, Canada

[†] Equal contribution

## Experimental Validation

### Excluded Influential Participant

Participants with missing responses exceeding two standard deviations above the mean in the dataset were excluded in the analyses. One participant in the Lee & Stark (20) dataset was short of the cutoff and was deemed an influential point in the analyses (the cutoff was 46.55, and the participant had 43 missing responses). We repeated the analyses after removing this participant.

### Sensitivity analysis - Semantic Perceptual Similarity derived from Deep Neural Networks

We sought to trial an alternative dissimilarity measure to lure bins to address a potential circularity induced by the fact that lure bins are used to determine discrimination ability, yet are themselves derived from human participants’ discrimination performance. Therefore, we conducted an additional analysis using the Wahlheim et al. [(32)](https://www.zotero.org/google-docs/?xC32Ew) dataset, employing deep neural networks to quantify the dissimilarity of test items in the MST. These models enable the transformation of raw images onto embeddings that represent images' perceptually salient high-level features.

To obtain image embeddings of MST images, we leveraged a deep learning model from the [*MetalHead.jl* package](https://fluxml.ai/Metalhead.jl/dev/) for the Julia programming language, *ResNet-152* [(34)](https://www.zotero.org/google-docs/?eqpoDc). This model was trained to recognize images from the ImageNet database. When using this trained model with MST images, we used the model’s highest-level representation of each image to compare the images with one another. Specifically, the model transformed each MST image into a continuous vector by extracting the model’s high-level embeddings. These embeddings can be thought of as high-level abstract representations of the contents of an image. With a vector representation of each MST image, we then defined the neural network-derived “perceptual dissimilarity” between image pairs as the cosine distance between their respective image vectors.

Since each participant did not study the same set of images in the study phase, we calculated each test trial’s dissimilarity independently for each participant. A given test trial image’s dissimilarity was thus defined as the neural network-derived “perceptual distance” between that test trial image and its least “perceptually dissimilar” studied image. A comparison between each trial image’s neural network-derived dissimilarity measure and the trial image’s lure bin is depicted in Figure S2. With this key difference in how dissimilarity is defined in the statistical analysis plan, the rest of the procedure was conducted as described in the Statistical analysis.

## Synthetic Data Experiments

### Spread analysis

To examine the robustness of the measures to the number of trials, additional simulations were conducted where the number of trials was systematically altered. At each set number of trials, 20 different sets of 50 agents with identical parameters were generated. Then, for each set of agents in which the agents have the same parameters, but through random discrete choices have variance in outcomes between them, we examined their variance in several outcome measures in relation to the number of trials the agents underwent. Like before, this analysis was repeated with a subsetted group of agents who had a Δ index of at least 0.6.

## Supplementary Results

Table S1 and Figure S3C show the results of the analysis on the collinearity of λ and Δ after excluding one participant due to substantial missing responses (albeit falling just below the 2 standard deviation threshold we pre-specified in our methods). After excluding this participant, the fixed effects coefficient was comparatively lower in magnitude (β=0.15, 95% CI [-0.08, 0.37]; p = 0.197), continuing to highlight the lack of linear association between λ and Δ.

Tables S2 and S3 outline our approach using mixed-effects modeling to examine the concordance between the original MST performance indices and our λ and Δ measures, after the exclusion of an influential participant with considerable missing responses. For our λ measure, the results reveal a significant association with the MST LDI index (fixed effects β=0.75, 95% CI [0.61, 0.90], p < 0.001; see Table S2 and Figure S3A). The Δ measure is not significantly associated with the MST LDI with a fixed effects coefficient of β=-0.05 (95% CI [-0.20, 0.10], p = 0.438; see Table S2 and Figure S3D). The association between λ and LDI remains highly consistent across studies, given a between-study variance of 0. Our Δ measure exhibited a significant association with the MST REC (fixed effects β=0.95, 95% CI [0.86, 1.04], p < 0.001; see Table S3 and Figure S3B). The λ measure is not significantly associated with the MST REC, showing a fixed effect coefficient of β=-0.00 (95% CI [-0.08, 0.08], p = 0.995; see Table S3 and Figure S3E). The association between Δ and REC demonstrated a marginal R^2^ of 0.852 and a conditional R^2^ of 0.866. An ICC of 0.09 suggests that this association was relatively consistent between the two studies.

The sensitivity analysis comparing linear models of LDI in the Lee & Stark [(27)](https://www.zotero.org/google-docs/?c31300) dataset with and without exclusion of the most similar lures in the λ and Δ calculations performed similarly to one another. The model without any lure exclusion (R^2^ = 0.826) demonstrated a slightly stronger R^2^ than the model with the excluded lures (R^2^ = 0.783; See Table S4). The sensitivity analysis of the Wahlheim et al [(32)](https://www.zotero.org/google-docs/?Xcx72t) dataset comparing neural network-derived dissimilarity measures with the standard MST lure bins was similar for both approaches in the association between the λ index and the LDI. When using neural network-derived distances as the dissimilarity measure, the relationship between the λ index and LDI demonstrated a slightly stronger explanatory power (R^2^ = 0.563, see Figure S4A and Table S5) compared to using lure bin values (R^2^ = 0.531).

Our investigation into the associations between various simulation parameters and the novel indices revealed the following: The Δ index is primarily associated with the parameters ρ (probability of recognizing old stimuli) and ψ (probability of remembering that an item was not studied), showing its sensitivity to changes in recognition memory performance (see Figure S6). The λ index showed a primary association with the parameter τ (the threshold for discriminability in relation to lure similarity), but it did not show a significant relationship with β (which determines the steepness of the logistic function relating discriminability to lure similarity), suggesting that λ's sensitivity is more attuned to the discriminability threshold than to variations in the steepness of discriminability across different levels of lure similarity (see Figure S7).

The spread analysis showed the variation in results that can be seen among identical synthetic agents in relation to the number of trials the agents underwent (see Figures S7 and S8). Without any participant exclusions, all measures except the λ index showed similar decreases in spread with increasing number of trials (Figure S8). The λ index in contrast demonstrated considerably less dropoff in spread with increasing trials. Interestingly, when subsetting the agents to allow only those with moderately high recognition (Δ index of at least 0.6; Figure S9), all measures across all number of trials showed less variation, and the λ index showed a similar dropoff to LDI with increasing trials.

## Supplementary Figures


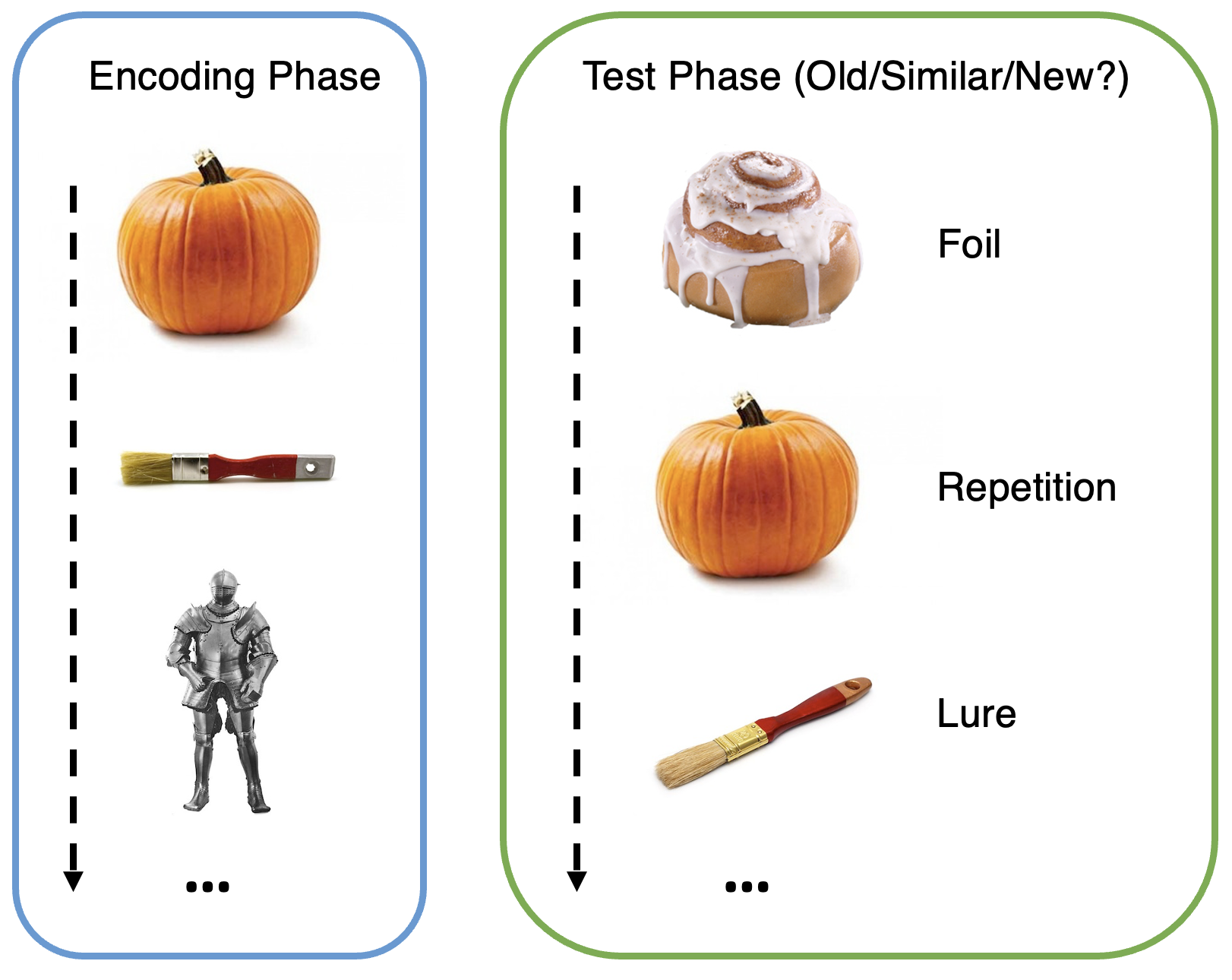


**Figure S1:** Examples of stimuli during the encoding and test phases of the Mnemonic Similarity Task.


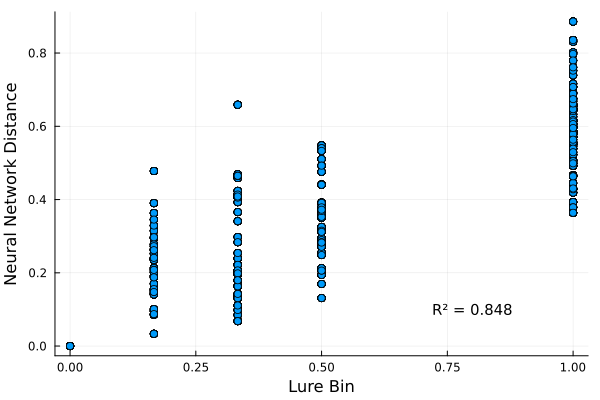


**Figure S2:** Neural network-derived distance measure compared to the original Lure Bin in the Wahlheim et al. (2021) MST dataset. Both measures are scaled to be between 0 and 1.

**
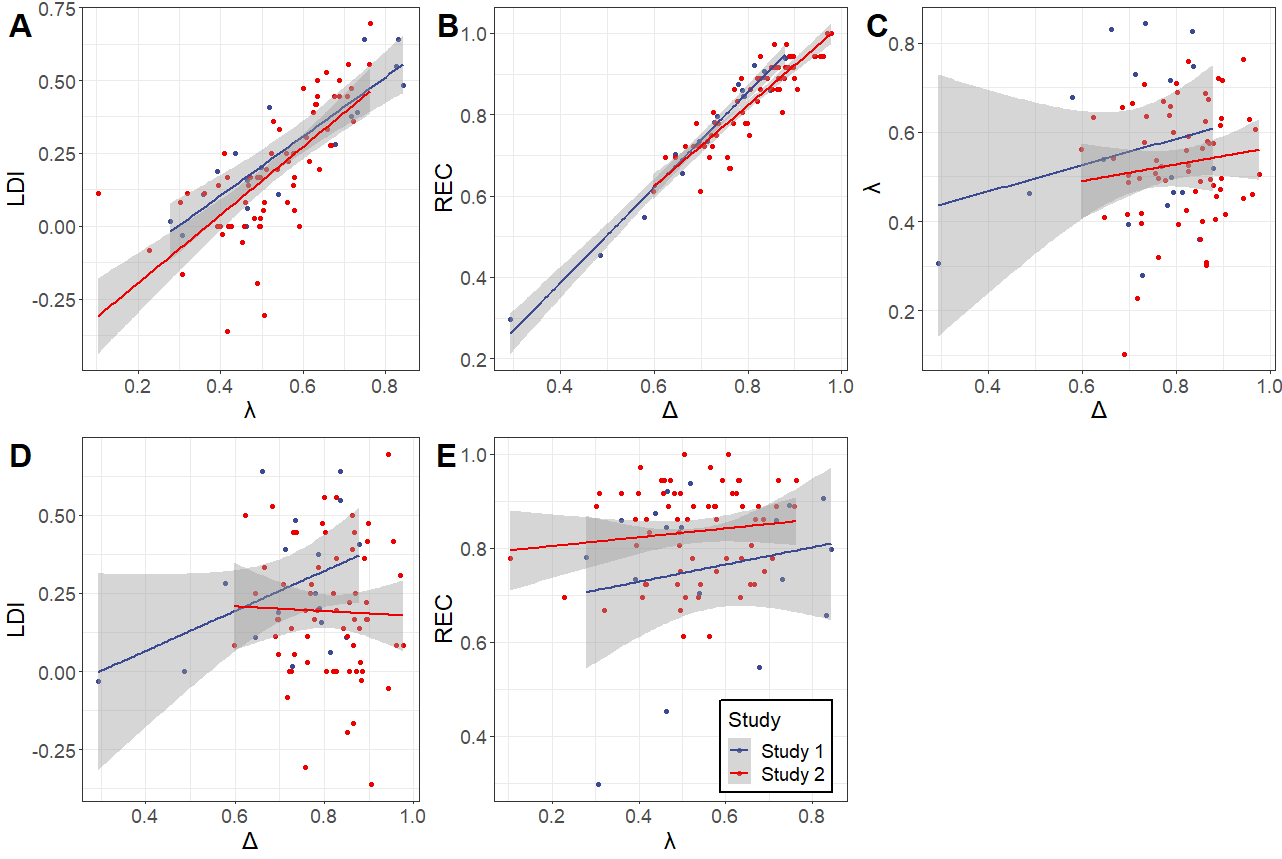
**

**Figure S3**: Pairwise comparisons of the original mnemonic similarity task (MST) measures and our novel measures with an additional participant excluded in the analysis due to considerable missing responses. Colors indicate the study the data is taken from: Lee & Stark 2023 in blue [(21)](https://www.zotero.org/google-docs/?mGOcZO), Wahlheim et al. 2021 in red [(26)](https://www.zotero.org/google-docs/?Jpv0rm). ***Panel A:*** The MST’s lure discrimination index (LDI) and the novel λ measure. ***Panel B:*** The MST’s recognition (REC) and the novel Δ measure. ***Panel C:*** The novel Δ and the novel λ measure. ***Panel D:*** The MST’s LDI and the novel Δ measure. ***Panel E:*** The MST’s REC and the novel λ measure.


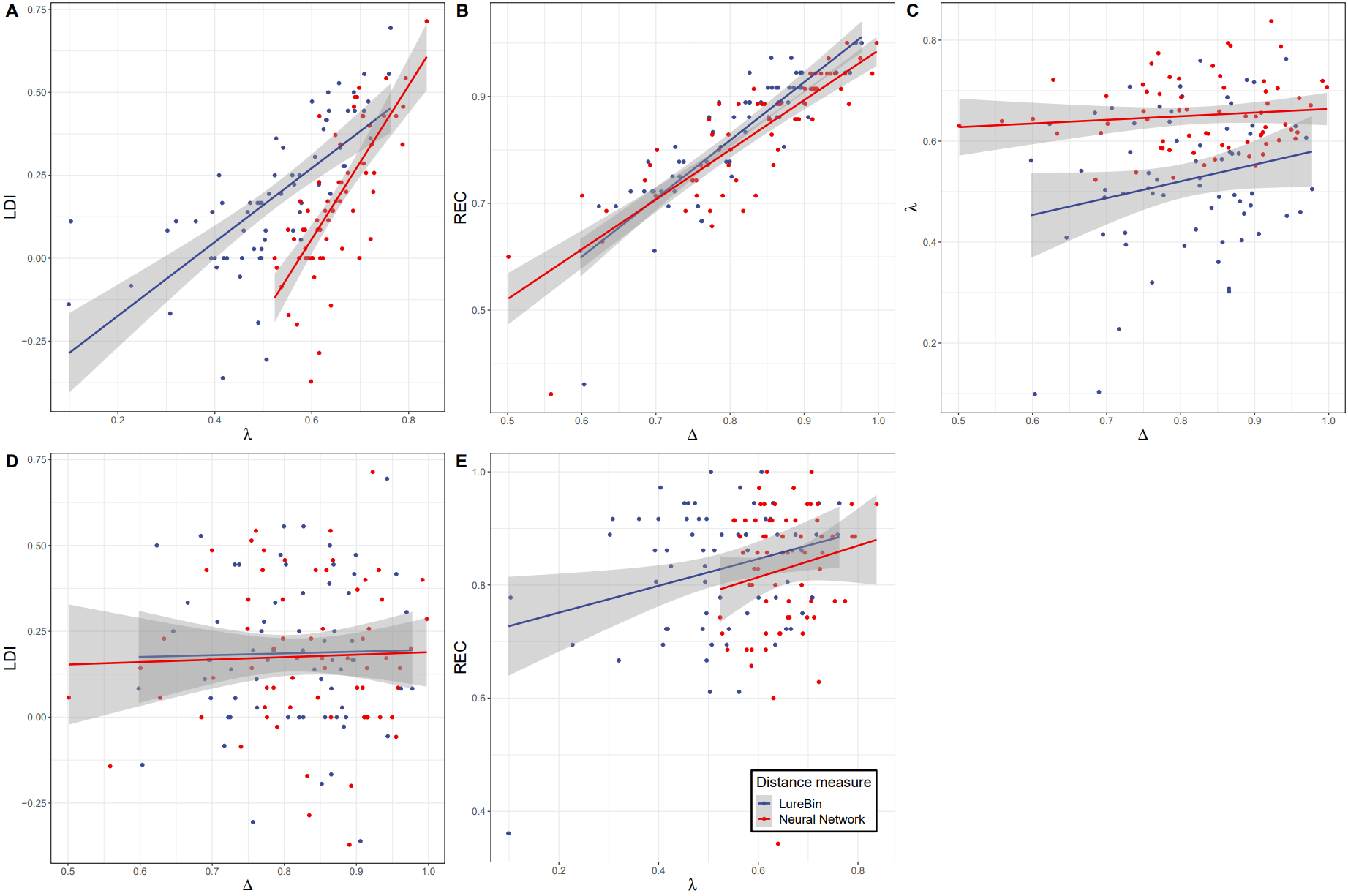


**Figure S4**: Pairwise comparisons of the original mnemonic similarity task (MST) measures and our novel measures using the Wahlheim et al. 2021 dataset using two different dissimilarity measures. Colors indicate the dissimilarity measure that was used to fit the logistic function. ***Panel A:*** The MST’s lure discrimination index (LDI) and the novel λ measure. ***Panel B:*** The MST’s recognition measure (REC) and the novel Δ measure. ***Panel C:*** The novel Δ and the novel λ measure. ***Panel D:*** The MST’s LDI and the novel Δ measure. ***Panel E:*** The MST’s REC and the novel λ measure.


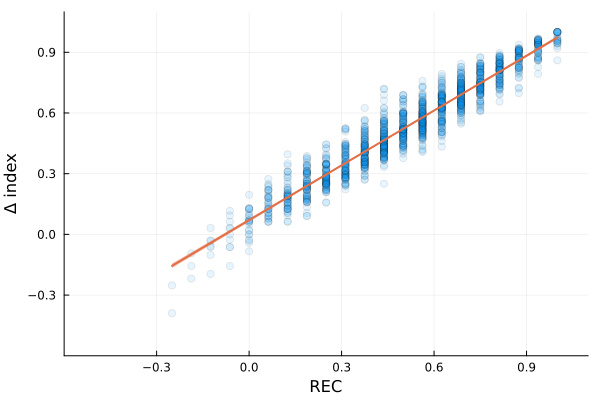


**Figure S5:** Relationship between the novel Δ index and the MST’s recognition measure (REC) in the synthetic data experiment.


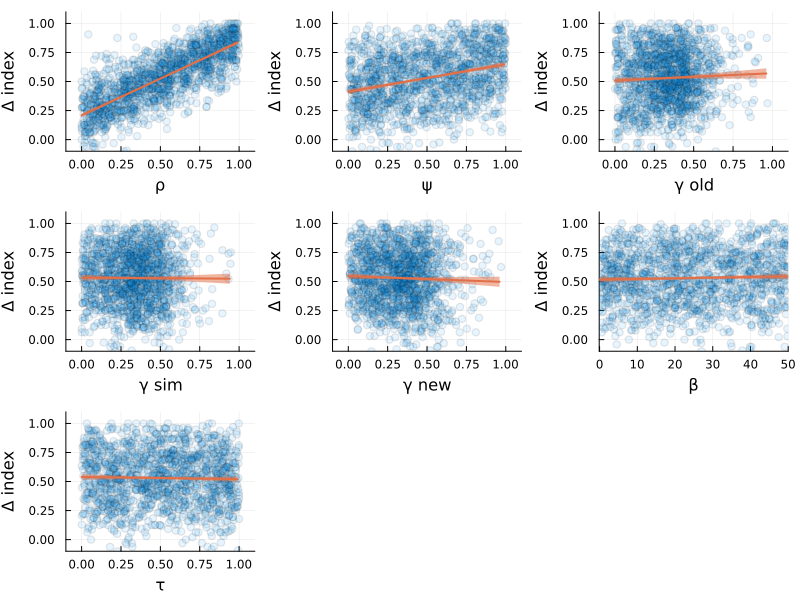


**Figure S6:** Associations between the Δ index and the synthetic agent parameters.


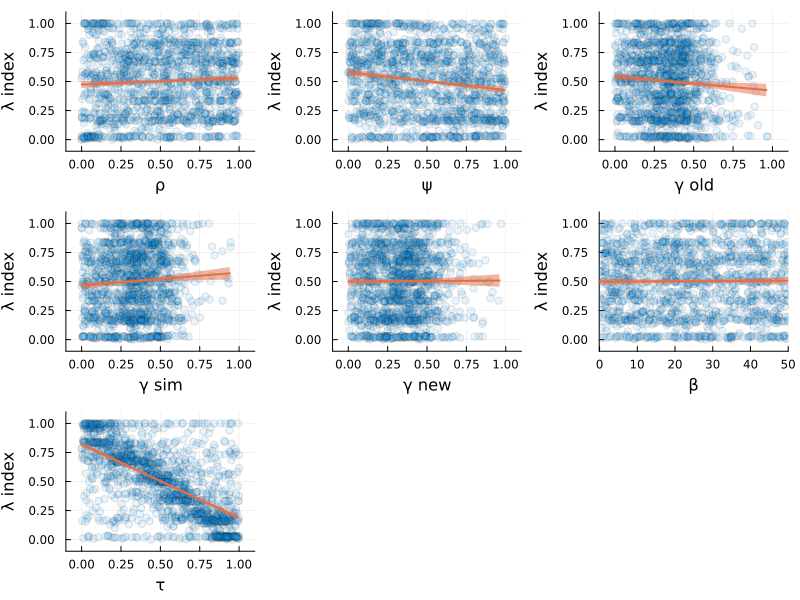


**Figure S7:** Associations between the λ index and the synthetic agent parameters.


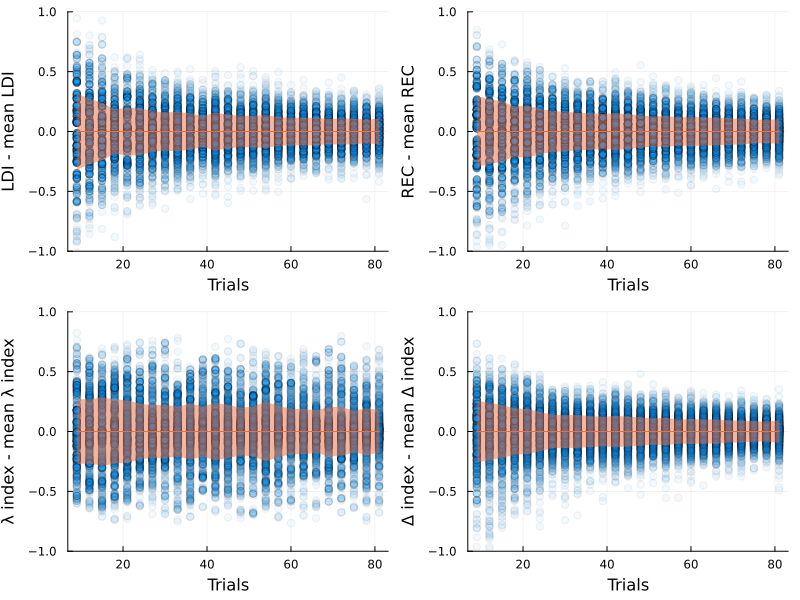


**Figure S8:** Variance of the LDI, REC, λ, and Δ measures in relation to the number of test trials undergone by synthetic agents. 50 copies of 20 unique synthetic agents underwent the simulated MST-like experiment at varying numbers of trials. The number of trials ranged from 6 to 81 at increments of 3. Each plot point represents a specific agent’s score compared to the average of its copies who underwent the same number of test trials. Ribbons show the standard deviation among the agents who underwent the same number of test trials.


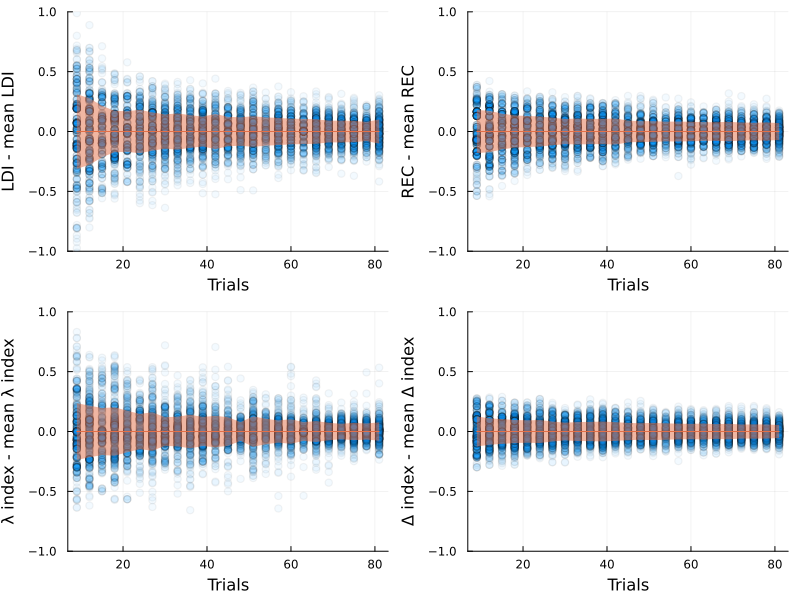


**Figure S9:** Variance of the LDI, REC, λ, and Δ measures in relation to the number of test trials undergone by synthetic agents with a Δ index of at least 0.6. 50 copies of 20 unique synthetic agents underwent the simulated MST-like experiment at varying numbers of trials, with those who obtained a Δ index below 0.6 removed. The number of trials ranged from 6 to 81 at increments of 3. Each plot point represents a specific agent’s score compared to the average of its copies who underwent the same number of test trials. Ribbons show the standard deviation among the agents who underwent the same number of test trials.

## Supplementary Tables

**Table S1**

*Mixed effects model results of Δ* *predicting λ scores with an additional participant excluded in the analysis due to considerable missing responses*

| Model 3: *λ ~ Δ + (1\|study)* |  | | |
| --- | --- | --- | --- |
| *Predictors* | *Estimates* | *CI* | *p* |
| Intercept | 0.04 | -0.29 - 0.38 | 0.794 |
| Δ | 0.15 | -0.08 - 0.37 | 0.197 |
| **Random Effects** |  |  |  |
| σ2 | 0.99 |  |  |
| τ00 study | 0.03 |  |  |
| ICC | 0.03 |  |  |
| N study | 2 |  |  |
| Observations | 84 |  |  |
| Marginal R^2^ / Conditional R^2^ | 0.021 / 0.049 |  |  |

**Table S2**

*Mixed effects model results of λ and Δ predicting original MST LDI scores with an additional participant excluded in the analysis due to considerable missing responses*

| Model 1: *LDI ~ λ + Δ + (1\|study)* |  | | |
| --- | --- | --- | --- |
| *Predictors* | *Estimates* | *CI* | *p* |
| (Intercept) | -0.00 | -0.15 – 0.15 | 1.000 |
| λ | 0.75 | 0.61 – 0.90 | **<0.001** |
| Δ | -0.05 | -0.20 – 0.10 | 0.438 |
| **Random Effects** | | | |
| σ^2^ | 0.45 | | |
| τ_00_ _study_ | 0.00 | | |
| N _study_ | 2 | | |
| Observations | 84 | | |
| Marginal R^2^ / Conditional R^2^ | 0.556 / NA | | |

**Table S3**

*Mixed effects model results of λ and Δ predicting original MST REC scores with an additional participant excluded in the analysis due to considerable missing responses*

| Model 2: *REC ~ λ + Δ + (1\|study)* |  | | |
| --- | --- | --- | --- |
| *Predictors* | *Estimates* | *CI* | *p* |
| (Intercept) | 0.04 | -0.16 – 0.24 | 0.675 |
| λ | -0.00 | -0.08 – 0.08 | 0.995 |
| Δ | 0.95 | 0.86 – 1.04 | **<0.001** |
| **Random Effects** | | | |
| σ^2^ | 0.14 | | |
| τ_00_ _study_ | 0.01 | | |
| ICC | 0.09 | | |
| N _study_ | 2 | | |
| Observations | 84 | | |
| Marginal R^2^ / Conditional R^2^ | 0.852 / 0.866 | | |

**Table S4**

*Linear models applied to the Lee & Stark (2023) dataset with the novel λ and Δ indices derived from the dataset with and without exclusion of the most similar lure trials (lure bins 1, 2, and 3). Note that both models use an LDI calculated without any lure exclusions.*

| Model: *LDI ~ λ + Δ ,* All lures present | | | | Model: LDI ~ λ + Δ, Lurebins 1-3 removed | | | |
| --- | --- | --- | --- | --- | --- | --- | --- |
| *Predictors* | *Estimates* | *CI* | *p* | *Predictors* | *Estimates* | *CI* | *p* |
| (Intercept) | 0.00 | -0.22 – 0.22 | 1.000 | (Intercept) | -0.00 | -0.25 – 0.25 | 1.000 |
| λ | 0.82 | 0.59 – 1.06 | **<0.001** | λ | 0.80 | 0.54 – 1.06 | **<0.001** |
| Δ | 0.24 | 0.01 – 0.48 | **0.044** | Δ | 0.27 | 0.00 – 0.53 | **0.047** |
| Observations 18 | | | | Observations 18 | | | |
| Marginal R^2^ / Conditional R^2^ : 0.826 / 0.803 | | | | Marginal R^2^ / Conditional R^2^ 0.783 / 0.754 | | | |

**Table S5**

*Linear models applied to the Wahlheim et al (2021) dataset using two different distance measures for lure stimulus: Lure Bin (left), Neural network-derived embeddings (right).*

| Model: *LDI ~ λ + Δ ,* Distance measure = Lure Bin | | | | Model: LDI ~ λ + Δ, Distance measure: Neural Net | | | |
| --- | --- | --- | --- | --- | --- | --- | --- |
| *Predictors* | *Estimates* | *CI* | *p* | *Predictors* | *Estimates* | *CI* | *p* |
| (Intercept) | 0.00 | -0.17 – 0.17 | 1.000 | (Intercept) | 0.00 | -0.16 – 0.16 | 1.000 |
| λ | 0.75 | 0.57 – 0.92 | **<0.001** | λ | 0.75 | 0.59 – 0.92 | **<0.001** |
| Δ | -0.14 | -0.32 – 0.03 | 0.111 | Δ | -0.05 | -0.21 – 0.12 | 0.574 |
| Observations 67 | | | | Observations 67 | | | |
| Marginal R^2^ / Conditional R^2^ : 0.531 / 0.517 | | | | Marginal R^2^ / Conditional R^2^ 0.563 / 0.549 | | | |
